# Prevalence of Sleep Inadequacy Among Badminton Players: A Systematic Review

**DOI:** 10.7759/cureus.87495

**Published:** 2025-07-08

**Authors:** Saranrat Manunyanon, Kanapot Pengked, Yuttachai Hareebin, Weeratian Tawanwongsri

**Affiliations:** 1 Center for Cultural and Sports Promotion, Walailak University, Nakhon Si Thammarat, THA; 2 Division of Sports Medicine, Department of Orthopedic Surgery, School of Medicine, Walailak University, Nakhon Si Thammarat, THA; 3 Faculty of Humanities and Social Sciences, Pattani Campus, Prince of Songkla University, Pattani, THA; 4 Division of Dermatology, Department of Internal Medicine, School of Medicine, Walailak University, Nakhon Si Thammarat, THA

**Keywords:** athletes, badminton, prevalence, risk factors, sleep deprivation, sleep quality, sports

## Abstract

Sleep quality is a critical determinant of athletic performance and recovery, yet limited research has specifically addressed this issue among badminton players. This systematic review aimed to estimate the prevalence of inadequate sleep and identify associated factors in this athletic population. Following a registered protocol (INPLASY202550010), a comprehensive literature search was conducted using the MEDLINE, Scopus, and DOAJ (Directory of Open Access Journals) databases through April 2025. Eligible studies were those that assessed sleep health using validated tools in badminton athletes. A total of two studies met the inclusion criteria, comprising 165 players. The prevalence of poor sleep health ranged from 28% to 36%. Factors contributing to suboptimal sleep included high weekly training volumes, delayed sleep onset, and academic-related stress. In contrast, prior injuries, number of rest days, and overall training frequency showed no significant associations with sleep quality. These findings suggest that both physical and psychological demands may adversely influence sleep in badminton players. Although the available data are limited, the evidence underscores the importance of addressing sleep health in this population. Further research using standardized methodologies and larger sample sizes is warranted to better characterize sleep patterns and inform targeted interventions that support optimal athlete well-being and performance.

## Introduction and background

Poor sleep quality is a widespread public health concern affecting populations globally. Approximately 20%-40% of individuals experience poor sleep quality [1,2]. It refers to disrupted or inconsistent sleep patterns, trouble falling or staying asleep, and daytime tiredness or reduced functioning as a result. Sleep quality is influenced by several factors, including age, sex, job-related stress, and existing health conditions [3-5]. Inadequate sleep significantly impairs athletic performance by reducing agility, endurance, reaction time, and cognitive functions such as attention and memory [6]. Moreover, sleep is critical for recovery by supporting muscle repair, immune function, and hormonal regulation, whereas poor sleep hinders recovery and increases the risk of overtraining [7]. Sleep deprivation also heightens injury risk, particularly musculoskeletal injuries, due to disrupted hormonal and immune regulation [8]. Additionally, sleep disturbances may exacerbate mental health conditions, such as anxiety, depression, and stress, further affecting athletic performance and overall well-being [9].

Although sleep is increasingly recognized as a vital component of athletic performance and recovery, research specifically focusing on badminton players remains limited. Badminton is one of the most popular sports globally, with approximately 200 million participants [10]. Its inclusion in the Olympic Games significantly enhanced international recognition and expanded its global fan base [11]. Nevertheless, most studies have focused on team or contact sports, leaving a gap in understanding the effects of poor sleep on athletes in individual high-speed racquet sports [12]. This gap is important given the unique physiological and psychological demands of badminton, including high-intensity intermittent activity, rapid decision-making, and frequent international travel, all of which can adversely affect sleep and circadian stability [13,14]. Furthermore, factors commonly linked to poor sleep quality-such as training schedules, competition-related stress, screen use, and mental health challenges-remain underexplored [14-16]. Therefore, a systematic review is warranted to consolidate the available evidence on the sleep health of badminton players. This review aimed to estimate the prevalence of sleep inadequacy among badminton players and to identify and synthesize the associated factors, thereby addressing existing knowledge gaps and informing sport-specific guidelines and future research directions.

## Review

Methodology

Eligibility Criteria

This systematic review was registered with the International Platform of Registered Systematic Review and Meta-Analysis Protocols (INPLASY) (Registration No. INPLASY202550010). The study was conducted in accordance with the ethical standards of the Declaration of Helsinki. The protocol was reviewed and approved with exemption status by the Walailak University Ethics Committee (Protocol No. WUEC-22-120-01). Studies reporting the prevalence of sleep inadequacy or poor sleep quality among badminton players, regardless of age, sex, or level of play, were eligible for inclusion. Eligible designs included observational studies such as cross-sectional, cohort, and case-control studies. Experimental studies were also considered if relevant baseline prevalence data were available. Only articles published in English were included, and the search covered all available literature up to April 2025. Any definition of sleep inadequacy was accepted, provided the study clearly described its measurement. Studies focusing solely on interventions without reporting baseline prevalence were excluded.

Information Sources and Search Strategy

**Figure 1:**
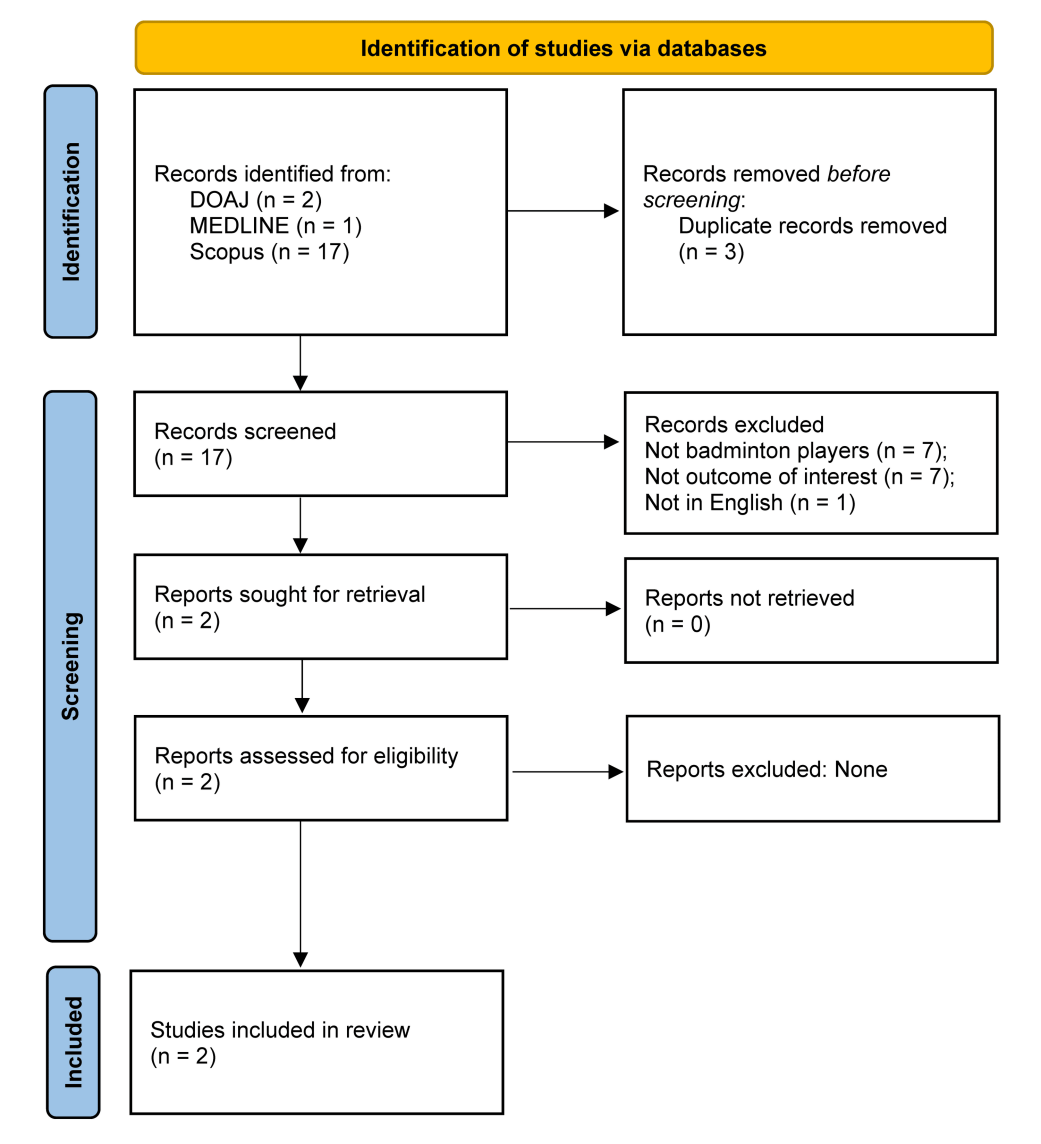
PRISMA flow diagram. DOAJ, Directory of Open Access Journals; MEDLINE, Medical Literature Analysis and Retrieval System Online; PRISMA, Preferred Reporting Items for Systematic Reviews and Meta-Analyses

A comprehensive literature search was conducted across MEDLINE, Scopus, and the Directory of Open Access Journals (DOAJ), covering all records from inception to April 2025. The search strategy combined Medical Subject Headings (MeSH) and free-text terms using Boolean operators (AND and OR) to optimize the retrieval of relevant studies. Key terms included “Racquet Sports”[MeSH], “badminton,” “Sleep Quality”[MeSH], “Sleep Initiation and Maintenance Disorders”[MeSH], “sleep inadequacy,” and related synonyms. The complete MEDLINE search string was: ("Racquet Sports"[MeSH] OR badminton) AND ("Sleep Quality"[MeSH] OR "Sleep Initiation and Maintenance Disorders"[MeSH] OR "sleep inadequacy" OR "sleep disturbance" OR "sleep disorder" OR "poor sleep"). Similar search strategies, adapted to the indexing systems of Scopus and DOAJ, were applied to ensure consistency across databases. The complete study selection process is illustrated in Figure 1.

Study Selection and Data Extraction

Titles and abstracts of all retrieved records were independently screened by two investigators to identify potentially eligible studies manually, without the use of automation tools or specialized software. Full texts of selected articles were reviewed in detail to determine final inclusion based on the predefined eligibility criteria. Discrepancies between the two reviewers were resolved through discussion, and a third reviewer was consulted if consensus could not be reached. Data extraction was performed independently by two investigators using a standardized data collection form. Extracted data included participant characteristics, details of badminton-related activity, prevalence of sleep inadequacy or poor sleep quality, sleep assessment tools used, and study location.

Risk of Bias and Methodological Quality Assessment

The methodological quality of the included studies was independently evaluated by two investigators using standardized tools appropriate for each study design. Discrepancies were resolved through discussion to reach consensus. Risk of bias was assessed using the Observational Study Quality Evaluation (OSQE) tool (Table 1) [17]. The overall risk of bias was determined based on how well each study met the OSQE criteria, the presence of any serious methodological flaws, and the clarity of reporting. Studies that met most quality criteria (approximately ≥75%), had no major issues, and demonstrated clear reporting were rated as having low risk of bias. Studies meeting some criteria (approximately 50%-75%), but without critical flaws, were categorized as moderate risk. Studies with at least one serious flaw indicated by a veto item were classified as high risk of bias, regardless of performance on other items. If key details were missing or reporting was unclear, the study was rated as having an uncertain risk of bias.

**Table 1:** Risk-of-bias assessment.

Authors (Year)	Design	Stars	Veto	Overall risk of bias
Wang et al. (2024) [18]	Repeated-measures observational study	9/16	None	Moderate risk
Skare et al. (2024) [19]	Cross-sectional study	7/10	None	Moderate risk

Software and Statistical Significance

No statistical software was used for this review. All data were descriptively synthesized based on the reported outcomes in the included studies. Statistically significant values (*P*-values and correlation coefficients) were extracted directly from the original articles.

Results

The final analysis included two studies, a cross-sectional study and a repeated-measures observational study, comprising 165 participants. The pooled, weighted mean age was 17.2 years. Most participants were men (*n* = 93, 56.4%). These studies were included after full-text screening and data extraction (Table 2).

**Table 2:** Studies on sleep disorders among badminton athletes. The ASBQ defines sleep behavior as “good” with a global score ≤ 36 and “bad” with a score ≥ 42. A PSQI score ≥ 5 was used to define poor sleep quality. An ESS score ≥ 10 indicates clinically elevated levels of daytime sleepiness. ASBQ, Athlete Sleep Behavior Questionnaire; ESS, Epworth Sleepiness Scale; PSQI, Pittsburgh Sleep Quality Index; SD, standard deviation

Authors (Year)	Study setting	Study design	Participant characteristics	Assessment tool(s)	Prevalence of sleep disorder/insomnia
Wang et al. (2024) [18]	China	Repeated-measures observational study	12 dual-career collegiate badminton athletes (mean age: 20.3 ± 1.7 years; 66.7% male)	PSQI ESS	Poor sleep quality (PSQI ≥ 5): 36.1%; mean PSQI score: 3.78 ± 2.39; daytime sleepiness (ESS ≥ 10): 25.0%; mean ESS score, 7.15 ± 5.48; mean sleep duration: 7 hours 15 minutes ± 61 minutes
Skare et al. (2024) [19]	Canada	Cross-sectional study	153 elite junior badminton players at the World Junior Championship (mean age: 17 years; SD not reported); 55.6% male	ASBQ	28% reported poor sleep behavior

Prevalence of Poor Sleep Health

The prevalence of poor sleep health ranged from 28.0% to 55.6% [18,19]. Skare et al. reported that 28% of 153 participants had poor sleep scores. There were no significant differences in sleep scores between men and women (*P* = 0.102), athletes aged <17 years and ≥17 years (*P* = 0.152), or Asian and non-Asian athletes (*P* = 0.062). Similarly, Wang et al. [18] found that approximately 36.1% of athletes reported poor sleep quality (Pittsburgh Sleep Quality Index (PSQI) score ≥5) over a six-month study period. Additionally, 25% of participants experienced excessive daytime sleepiness (ESS score ≥10).

Factors Associated with Poor Sleep Quality

Skare et al. [19] reported no significant differences in sleep scores based on a history of significant injuries (*P *= 0.158). Similarly, training load was not associated with sleep scores (*P *= 0.476). The number of rest days also showed no significant differences in sleep scores (*P* = 0.644). Wang et al. [18] found that several training-related and psychological factors were associated with sleep quality in dual-career collegiate badminton players. Greater weekly training hours were associated with poorer sleep quality, particularly in July (*r *= 0.573; *P *= 0.026). Athletes typically trained three to five times per week for approximately 1.5 hours per session. Although the timing of training sessions was not recorded, the average bedtime of approximately 11:52 PM suggested that late training may have contributed to delayed sleep onset. Although the number of competitions did not significantly affect sleep quality, it was positively associated with increased daytime sleepiness in August (*r *= 0.598; *P* = 0.040). Academic stress emerged as a key psychological factor, with weekly study hours significantly correlated with poor sleep quality (*r* = 0.308; *P* = 0.009). This association was particularly strong in August (*r* = 0.868; *P *< 0.001), highlighting the impact of academic workload on sleep quality in athletes.

Discussion

To our knowledge, this is among the first systematic reviews to focus specifically on sleep health in badminton players, offering a comprehensive synthesis of current evidence in an understudied athletic population. The review was conducted using a registered protocol (INPLASY) and involved a systematic search across multiple databases to ensure comprehensive coverage of relevant studies. Additionally, a structured risk-of-bias tool (OSQE) was used to assess methodological quality, enhancing the rigor and transparency of the findings. This review highlights that a substantial proportion of badminton players experience inadequate sleep, with prevalence rates ranging from 28% to 36%. Although the included studies varied in methodology and assessment tools, their findings were generally consistent, indicating that sleep-related issues are common among badminton players. Key contributing factors include high weekly training volumes, delayed bedtimes, and academic pressure, particularly among dual-career athletes. Conversely, previous injuries, rest duration, and general training frequency were not significantly associated with sleep quality [18, 19]. These findings underscore the multifactorial nature of sleep disturbances in badminton athletes, shaped by both physiological demands and psychosocial stressors.

This issue is prevalent among athletes, who are often perceived as physically resilient but remain vulnerable to distinct psychological and physiological stressors. Swinbourne et al. [20] conducted a cross-sectional study using self-reported questionnaires to assess sleep quality, daytime sleepiness, and sleep apnea risk in 175 elite athletes aged ≥18 years from national teams and regional squads. Participants were from Rugby Union, Rugby Sevens, and Cricket. Half were classified as poor sleepers (PSQI > 5), with a mean score of 5.9 ± 2.6; 65% scored ≥5, 22% ≥8, and 9% ≥10, indicating widespread sleep disturbances. Hoshikawa et al. [21] conducted a similar cross-sectional study to assess sleep quality among elite Japanese athletes. Of the 891 athletes initially enrolled, data from 817 (91.7%) aged >20 years were analyzed. All were candidates for the 17th Asian Games in Incheon 2014, including 449 men and 368 women, representing various sports such as aquatics, athletics, judo, and football. Poor sleep quality (PSQI >5.5) was identified in 28.0% of participants (229/817). Mah et al. [22] administered a questionnaire-based survey to evaluate sleep quality among 628 collegiate athletes at Stanford University (343 men and 285 women; mean age, 19.6 ± 1.3 years). Athletes represented 29 varsity sports, including individual and team events such as basketball, rowing, soccer, swimming, and wrestling. Results showed that 42.4% were classified as poor sleepers (PSQI > 5), and 104 athletes (16.6%) reported *fairly bad *or *very bad* sleep quality, with a higher prevalence among men (19.5%) than women (13.0%). More recently, Rebello et al. [23] conducted a cross-sectional descriptive study to examine sleep behaviors and characteristics among varsity athletes. The study included 64 participants (29 men and 34 women; mean age, 20.3 ± 1.7 years), with the majority (71%) classified as Caucasian participants. Athletes participated in various sports, including soccer (41%), rugby (17%), basketball (13%), volleyball (13%), track and field/cross-country (8%), and hockey (8%). The results showed that 37% had moderate-to-severe clinical sleep problems based on the Athlete Sleep Screening Questionnaire, and 62% demonstrated poor sleep behaviors based on the Athlete Sleep Behavior Questionnaire. Only 51% met the recommended minimum of ≥7 to 9 hours of sleep per night.

While previous research has documented poor sleep quality among athletes in various sports, including rugby, cricket, swimming, and basketball [20], the present review demonstrates that badminton players are comparably affected. Despite the global popularity of badminton and its unique combination of high-speed, intermittent exertion, and rapid decision-making [10], it remains underrepresented in sleep-related literature. The consistency of prevalence rates across different sports reinforces the notion that sleep inadequacy is not confined to high-contact or endurance-based activities [15,24]. Therefore, greater attention should be given to sleep monitoring and recovery strategies in badminton to support athlete health and optimize performance outcomes.

Across athletic populations, several shared factors have consistently been associated with poor sleep quality. One prominent factor is the use of electronic devices before bedtime, which negatively impacts sleep hygiene and increases sleep latency [18,21]. Psychological stressors, including depressive symptoms and emotional distress, are also common contributors to sleep disturbances. Multiple studies have linked mood-related symptoms and academic stress to impaired sleep quality [18,21,23]. In addition, disrupted sleep due to demanding training schedules and competitive travel is a well-documented issue across athlete cohorts [19,22]. Inadequate sleep duration, particularly sleeping fewer than 7 hours per night, is frequently reported and associated with poorer sleep quality and increased daytime sleepiness [18,22]. This concern may be further exacerbated in younger athletes, such as those with an average age of 17.2 years, as adolescents typically require more sleep than adults to support cognitive and physical development. Two recent badminton-specific studies provide insight into sport-specific sleep challenges. A study demonstrated that academic workload and weekly training hours were significant predictors of poor sleep quality among collegiate athletes balancing sports and education [18]. Furthermore, fluctuations in competition and examination schedules were found to influence sleep, although these effects were less consistently observed. In another study, Skare et al. [19] observed a high prevalence of poor sleep behavior and musculoskeletal symptoms among junior elite players, though no statistically significant association was identified between the two. Nonetheless, the authors highlighted the potential impact of travel disruptions, competition-related stress, and physical discomfort on sleep patterns. Additionally, concerns related to athletic identity and performance pressure were noted as relevant psychological stressors in this context [18]. Overall, these findings emphasize the multifactorial nature of sleep challenges in badminton players, particularly those navigating the dual demands of academic and athletic commitments.

Sleep is broadly classified into two main types: non-rapid eye movement (NREM) sleep and rapid eye movement (REM) sleep [25]. A typical night comprises approximately four to six sleep cycles, each lasting between 90 and 120 minutes. Within each cycle, both NREM and REM stages occur, with the proportion of REM sleep increasing in the latter half of the night. For the general adult population, it is recommended to obtain between seven and nine hours of sleep per night to maintain optimal health and daily functioning [26,27]. This recommendation is grounded in the necessity of completing multiple sleep cycles to ensure sufficient time in both NREM and REM phases. NREM is essential for physical restoration, including tissue repair, immune system support, and energy replenishment. Meanwhile, REM sleep contributes significantly to memory consolidation, learning processes, and emotional regulation [28,29]. Furthermore, consistent and sufficient sleep has been linked to a lower risk of developing chronic health conditions such as cardiovascular disease, obesity, and diabetes [30]. For athletes, the standard sleep recommendation of seven to nine hours per night may not be sufficient. Due to the physical and cognitive demands of training and competition, athletes often require additional sleep to promote recovery and enhance performance [31,32]. In our review, one study reported that athletes slept an average of seven hours and 15 minutes, which falls below the optimal threshold often suggested for athletic populations [18]. This shortfall may compromise their physical restoration, cognitive function, and overall readiness. Therefore, tailored sleep strategies that consider individual needs, training intensity, and recovery status are essential to ensure athletes achieve sufficient sleep for peak performance.

This systematic review has several limitations that must be acknowledged. First, only two studies met the inclusion criteria, encompassing a combined sample of 165 participants. The limited number of studies and uneven sample sizes reduced the statistical power and constrained the generalizability of the findings. These results should, therefore, be interpreted as preliminary, and caution is warranted in drawing broad conclusions or applying them to wider athletic populations. Second, the included studies utilized different sleep assessment tools (ASBQ, PSQI, and ESS), evaluated conceptually distinct outcomes, and investigated heterogeneous populations ranging from elite junior athletes to dual-career collegiate players. These methodological and clinical variations introduced substantial heterogeneity, making meta-analysis inappropriate. Moreover, the use of different tools may yield varying prevalence estimates, complicating direct comparisons across studies and potentially affecting the interpretation of overall sleep inadequacy trends. Third, the review did not include gray literature or non-English-language sources. This may have resulted in the omission of potentially relevant studies, particularly from regions where badminton is highly popular but research may not be published in peer-reviewed English-language journals. This exclusion may limit the comprehensiveness and global applicability of the findings. Future research should incorporate longitudinal designs and objective sleep-monitoring methods to enhance the reliability and validity of outcomes. Intervention trials tailored to badminton athletes are warranted, particularly those targeting modifiable factors such as training schedules, academic stress, and sleep hygiene practices. In addition, the effectiveness of behavioral strategies, including cognitive-behavioral therapy for insomnia, mindfulness-based stress reduction, and structured napping, should be investigated for their potential to enhance sleep quality and recovery [33,34]. Research efforts should aim to recruit larger and more diverse athlete populations across different competitive levels and regions. Disaggregating data by sex, playing level, and seasonal phase may help identify subgroup-specific risk factors. Furthermore, qualitative studies exploring athletes’ perceptions and attitudes toward sleep and recovery could provide valuable context-specific insights to inform tailored interventions. Standardizing sleep assessment tools and outcome definitions is essential for improving cross-study comparability and facilitating future meta-analyses.

The findings of this review offer practical guidance for coaches, sports medicine professionals, and athletic trainers working with badminton players. Given the prevalence of sleep disturbances in this population, incorporating sleep hygiene education into regular training routines is recommended. Particular attention should be paid to managing the academic demands faced by dual-career athletes, optimizing training schedules to minimize disruptions in circadian rhythms, and reducing screen exposure before bedtime. Personalized recovery strategies, including the use of wearable devices to monitor sleep patterns, may aid in the early identification of sleep-related issues. Finally, fostering collaboration among coaches, psychologists, and sleep health professionals can help create a more supportive environment that promotes both athletic performance and overall well-being.

## Conclusions

This systematic review underscores the high prevalence of sleep inadequacy among badminton athletes, with reported rates ranging from 28% to 36%. Although based on a limited number of studies, the findings suggest that sleep disturbances are a common concern in this population and are influenced by factors such as training volume, academic stress, and late bedtimes. However, variability in sleep assessment tools, outcome definitions, and participant characteristics highlights the need for greater methodological consistency in future research. Addressing these gaps will improve the understanding of sleep health in badminton athletes and support the development of targeted interventions to enhance both athletic performance and overall well-being.
